# The effect of tourniquet application on optic nerve sheath diameter and cerebral oxygenation during lower extremity surgery: A prospective observational study

**DOI:** 10.1097/MD.0000000000047789

**Published:** 2026-02-20

**Authors:** Bahar Aktaş, Rahşan Dilek Okyay, Özcan Pişkin, Bengü Gülhan Köksal İncegül, Gamze Küçükosman, Eren Açikgöz, Çağdaş Baytar, Keziban Bollucuoğlu, Hilal Ayoğlu

**Affiliations:** a Department of Anesthesiology and Reanimation, Yalova Training and Research Hospital, Yalova, Turkey; b Department of Anesthesiology and Reanimation, Zonguldak Bülent Ecevit University, Faculty of Medicine, Zonguldak, Turkey; c Department of Anesthesiology and Reanimation, University of Health Sciences, Trabzon Faculty of Medicine, Trabzon, Turkey; d Department of Anesthesiology and Reanimation, Ereğli State Hospital, Zonguldak, Turkey.

**Keywords:** cerebral oxygenation, intracranial pressure, optic nerve sheath diameter, tourniquet

## Abstract

Tourniquet deflation during lower extremity surgery is associated with abrupt physiological changes that may influence intracranial pressure (ICP) and cerebral oxygenation. This prospective observational study aimed to evaluate changes in ICP following tourniquet deflation using ultrasonographic optic nerve sheath diameter (ONSD) measurements and to investigate their association with cerebral oxygenation parameters. This prospective observational study included 43 adult patients undergoing lower extremity surgery with tourniquet application under standardized general anesthesia. In addition to routine hemodynamic monitoring, bilateral cerebral oxygen saturation (rSO_2_) was continuously monitored using near-infrared spectroscopy, and ONSD measurements were performed at predefined time points. End-tidal carbon dioxide (EtCO_2_) levels and tourniquet duration were recorded. Patients were stratified into 2 groups according to ONSD values (≥5 mm and <5 mm), and rSO_2_, EtCO_2_, and tourniquet times were compared between groups. Correlation analyses were conducted to assess the relationships among these variables. ONSD and EtCO_2_ values measured at 5 and 15 minutes after tourniquet deflation were significantly higher than pre-deflation values. Bilateral rSO_2_ values following tourniquet deflation were significantly higher compared with measurements obtained before anesthesia induction, tourniquet inflation, and immediately prior to deflation. At 5 minutes after tourniquet deflation, EtCO_2_ levels and tourniquet duration were significantly higher in patients with ONSD ≥5 mm compared with those with ONSD <5 mm. Moderate, positive, and statistically significant correlations were observed between EtCO_2_ and ONSD, EtCO_2_ and bilateral rSO_2_, and between ONSD and tourniquet duration at this time point. Tourniquet deflation during lower extremity surgery is associated with transient increases in EtCO_2_, ONSD, and cerebral oxygenation parameters. These findings suggest significant physiological associations between tourniquet duration, carbon dioxide levels, and surrogate markers of ICP and cerebral oxygenation; however, further studies incorporating direct ICP measurements and clinical neurological outcomes are required to determine their clinical significance.

## 1. Introduction

Pneumatic tourniquets are frequently used in orthopedic extremity surgeries because they provide a clean surgical site and reduce blood loss. Despite the advantages of tourniquet use, several cardiovascular, metabolic, cerebral, and hemodynamic changes occur during the tourniquet inflation and deflation phases.^[1]^ As a tourniquet is deflated, the mean arterial pressure (MAP) and central venous pressure values decrease while the P_a_CO_2_ and end-tidal carbon dioxide (EtCO_2_) levels increase.^[2]^ With its strong vasoactive activity, P_a_CO_2_ plays an important role in the modulation of cerebral vasomotor tone. An increase in P_a_CO_2_ results in cerebral vasodilation and an increase in intracranial pressure (ICP).^[3]^ While intraventricular devices are the gold standard for ICP monitoring, such equipment is expensive and inconvenient because neurosurgeons who can use these devices may not always be available. Their use can also lead to complications, such as infection and bleeding. ICP can be measured noninvasively via optic nerve sheath diameter (ONSD) ultrasonography. The optic nerve forms part of the central nervous system and is surrounded by cerebrospinal fluid. Changes in ICP are reflected in the subarachnoid space, especially the retrobulbar space. An increase in pressure causes an increase in the ONSD. For this reason, ONSD measurement is effective for detecting elevated ICP.^[4]^

Cerebral hypoperfusion, which is caused by the hypotension that occurs after tourniquet deflation, may lead to postoperative cognitive dysfunction.^[5]^ Near-infrared spectroscopy (NIRS) is a noninvasive and continuous monitoring tool that shows cerebral balance in the form of the ratios of oxygenated and deoxygenated hemoglobin in cerebral tissues. In this respect, NIRS is a trend monitor that can be used to prevent undesirable neurological events, such as cerebral hypoperfusion, in patients under anesthesia.^[6,7]^

In our study, we aimed to demonstrate potential changes in regional cerebral oxygen saturation (rSO_2_) and ONSD values following tourniquet inflation and deflation and to investigate the relationships between these variables.

## 2. Materials and methods

This prospective observational study was carried out in the operating rooms of the Department of Anesthesiology and Reanimation between May 1, 2019, and March 1, 2020, after obtaining approval from the Zonguldak Bülent Ecevit University Faculty of Medicine Non-Invasive Clinical Studies Ethics Committee (April 4, 2019, meeting/decision no.: 2019/05), registering the trial on ClinicalTrials.gov (no. NCT04758091), and securing written consent from the participating patients. The study sample included 43 patients who were scheduled to undergo elective lower extremity surgery under general anesthesia, were at least 18 years old, and had an American Society of Anesthesiologists (ASA) physical status of I to II.

The sample excluded patients who were undergoing emergency surgeries and those with a known neurological disease, carotid artery disease, a history of intracranial or ocular surgery, neurological symptoms, known cerebral edema, high ICP, glaucoma, a body mass index (BMI) > 35 kg/m^2^, uncontrolled hypertension, diabetic retinopathy, anemia, coronary artery disease, peripheral artery disease, hemodynamic instability, pulmonary and/or surgical complications, or a tourniquet time of <60 or >120 min.

The sex, age, BMI value, ASA score, smoking status, comorbidities, and operation type were recorded for all the included patients. For each patient who was taken to the operating room without premedication, venous access was achieved using an 18 to 20-gauge catheter, and a crystalloid infusion was initiated. In addition to routine anesthesia monitoring (electrocardiography, noninvasive blood pressure, pulse oximetry, and EtCO_2_), the patients were monitored using the bispectral index, bilateral NIRS, and pleth variability index methods. The baseline monitoring and ONSD values were recorded. Preoxygenation was provided to each patient by supplying 100% O_2_ via a facemask for 3 minutes. For anesthesia induction, 1 mg/kg lidocaine (Aritmal 2%, Osel, Turkey), 1 mcg/kg fentanyl citrate (Fentanyl Citrate, Abbott Lab., North Chicago), 2 mg/kg propofol (Propofol-PF 1%, Polifarma, Turkey), and 0.6 mg/kg rocuronium bromide (Muscuron, Kocak Pharma, Turkey) were administered intravenously. For anesthesia maintenance, to ensure that the bispectral index would be in the range of 40 to 60, an infusion of 0.05 to 0.2 mcg/kg/min remifentanil hydrochloride (Ultiva, Eczacibaşi, Turkey) was administered, and sevoflurane at a minimal alveolar concentration of 0.8 to 1.2 (Sevorane, AbbVie, Queenborough, UK) was used in a 4-L 50% oxygen/air mixture. The patients were ventilated in volume-controlled mode at an 8 mL/kg tidal volume, 1:2 inspiratory/expiratory ratio, 12 to 14/min frequency, and 5 cm H_2_O positive end-expiratory pressure. It was decided to raise the respiratory rate if the EtCO_2_ was >45 mm Hg. After the extremity scheduled for operation had been elevated and venous return achieved, a tourniquet was applied at a pressure of 300 mm Hg.

Bleeding and fluid levels were monitored, and crystalloid fluids were replaced so that pleth variability index values would be <14. The patients were kept warm using overbody forced-air warming blankets, and their body temperatures were measured using a noncontact infrared thermometer 1 hour after anesthesia induction. The operating room temperatures were set at 22 °C.

The heart rate, MAP, peripheral oxygen saturation, ONSD, NIRS, and EtCO_2_ values of the patients were measured and recorded at certain times throughout the operation: before anesthesia induction (T0), right before tourniquet application (T1), 60 minutes after tourniquet application (T2), right before tourniquet deflation (T3), 5 minutes after deflation (T4), 15 minutes after deflation (T5), and 30 minutes after deflation (T6). Additionally, the volumes of fluids given, urinary output, total bleeding volumes, the types and volumes of the blood produsts given, tourniquet time, surgery time, anesthesia time, and pre- and postoperative hemoglobin values were recorded.

**Figure 1.**
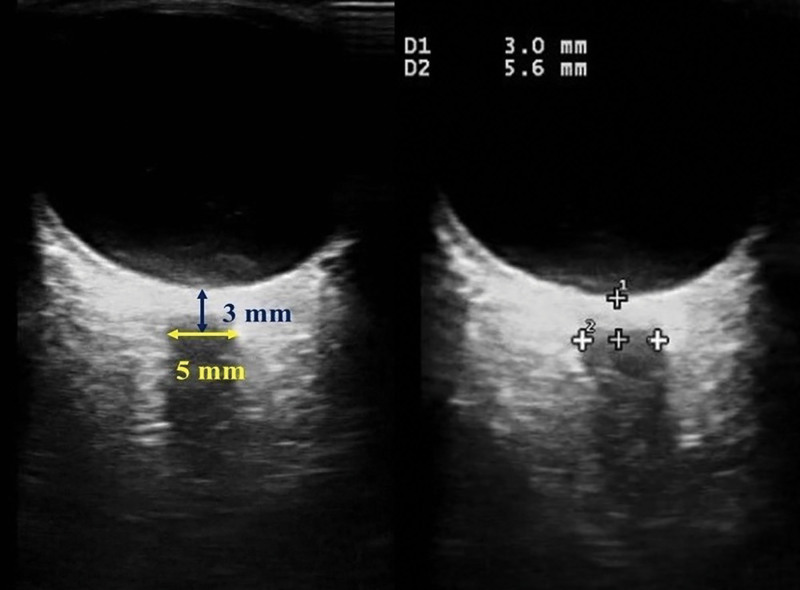
Optic nerve sheath diameter ultrasound image.

The ONSD measurements by ultrasound were performed by the same anesthesiologist who conducted the study; the anesthesiologist had prior experience with >25 ultrasonographic ONSD examinations. With the patient in the supine position and eyes closed, a thin layer of ultrasound gel was applied, and a linear 7.5 MHz ultrasound probe was gently placed on the upper eyelid without exerting pressure. After obtaining the optimal image between the retrobulbar echogenic adipose tissue and the vertical hypoechoic band in two-dimensional mode, the ONSD was measured 3 mm posterior to the papilla using an electronic caliper (Fig. 1). Two measurements were obtained in both the sagittal and transverse planes for each eye, and the mean of the 4 values was recorded as the ONSD value for that time point.

An ONSD threshold of ≥5.0 mm was used to indicate increased intracranial pressure. This cutoff value has been widely reported in the literature as a reliable surrogate marker of elevated ICP in adult patients, demonstrating high sensitivity and specificity when compared with invasive ICP monitoring techniques.^[8]^ Previous clinical and imaging studies have shown that an ONSD value of approximately 5.0 mm correlates with an ICP ≥20 mm Hg, and this threshold has been commonly adopted in perioperative and critical care settings.^[9]^

The recorded data were compared across the predefined time points. Based on ONSD measurements obtained after tourniquet deflation, patients were stratified into 2 groups: those with increased ICP (ONSD ≥ 5 mm) and those without increased ICP (ONSD < 5 mm). The groups were compared in terms of demographic characteristics, ASA physical status, BMI, smoking status, mean arterial pressure, EtCO_2_, rSO_2_, and tourniquet duration. Correlation analyses were performed to assess the relationships between EtCO_2_, ONSD, rSO_2_, and tourniquet time.

### 2.1. Statistical analysis

The sample size required to conduct the study was calculated as 35 for a 95% confidence interval and 80% power using the Power Analysis and Sample Size software, version 11 (NCSS, LLC, Kaysville), in line with the study conducted by Beşir and Tuğcugil.^[10]^ The calculated minimum sample size was increased by approximately 20%, and the sample included 43 patients.

The collected data were uploaded to a computer and analyzed using the Statistical Package for the Social Sciences for Windows program, version 22.0 (IBM, Armonk). The descriptive statistics are presented as the mean, standard deviation, minimum, and maximum values for the numeric variables and frequency and percentage values for the categorical variables. The Shapiro–Wilk test was used to examine whether the data were normally distributed. Paired-samples *t* tests were conducted to identify differences between the intragroup repeated measurements, while independent samples *t* tests were undertaken to identify differences between the 2 groups. Chi-squared tests (Yates-corrected) were performed to determine the significance of the differences between the categorical variables. Pearson correlation analysis was used to measure the relationships between the normally distributed quantitative variables. The level of statistical significance was established as *P* < .05.

## 3. Results

Two patients were excluded from the study because their tourniquet times were shorter than 60 minutes, and the study was completed with 41 patients aged 20 to 84 years. The demographic data and intraoperative values of the patients are shown in Table 1.

**Table 1:** Patients’ demographics and intraoperative values (n: 41).

	Mean ± SD
Gender (female/male)	19/22
Age (yr)	47.70 ± 17.16
Body mass index (kg/m^2^)	27.94 ± 3.54
ASA (I/II)	10/31
Current smoker (yes/no)	16/25
Anesthesia time (min)	183.14 ± 21.60
Surgery time (min)	171.65 ± 21.57
Tourniquet time (min)	112.80 ± 6.73

ASA = American Society of Anesthesiologists; SD = standard deviation.

In Table 2, the heart rate, MAP, EtCO_2_, ONSD, and right/left NIRS values of the patients at different measurement times are presented. The MAP values were significantly lower at all time points compared to the values measured before induction (*P* < .001). The MAP values measured after tourniquet inflation were higher than those measured immediately before tourniquet inflation (*P* < .05). The EtCO_2_ values measured after tourniquet deflation were higher than those measured before tourniquet inflation and before tourniquet deflation (*P* < .05). All the intraoperative ONSD values of the patients were higher than those prior to induction (*P* < .001). The ONSD values obtained 5 and 15 minutes after tourniquet deflation were higher than those obtained before deflation (*P* < .001).

**Table 2:** Patients’ heart rate, mean arterial pressure, EtCO2, ONSD, right and left NIRS values.

	T0	T1	T2	T3	T4	T5	T6
HR	77.78 ± 13.74	67.82 ± 11.29*	65.68 ± 10.47*	68.04 ± 10.32*	67.19 ± 10.10*	65.80 ± 9.54*,∥	66.58 ± 8.59*
MAP	100.04 ± 12.33	78.97 ± 10.44*	88.09 ± 10.43*,§	91.09 ± 10.19*,§	72.09 ± 7.16*,‡,∥	74.12 ± 8.36*,‡,∥	80.53 ± 9.50*,∥
EtCO_2_		32.97 ± 2.36	33.53 ± 2.27§	34.14 ± 2.31§	41.02 ± 2.61§,¶	36.90 ± 2.39§,¶	34.43 ± 1.87§
ONSD	3.71 ± 0.18	3.84 ± 0.22†	3.93 ± 0.18†,§	4.01 ± 0.16†,§	4.95 ± 0.21†,§,¶	4.23 ± 0.23†,§,¶	3.97 ± 0.19†,§
Right NIRS	65.53 ± 5.01	67.43 ± 6.14†	66.60 ± 6.71†,§	68.04 ± 6.34†,§,¶	69.41 ± 7.49†,§,¶	70.17 ± 6.68†,§,¶	69.19 ± 5.60†,§,¶
Left NIRS	65.17 ± 5.43	65.82 ± 6.71	66.00 ± 7.55	66.78 ± 7.43	68.19 ± 7.93†,§,¶	69.17 ± 6.90†,§,¶	68.14 ± 6.09†,§,¶

T0 = before anesthesia induction, T1 = right before tourniquet application, T2 = 60 minutes after tourniquet application, T3 = right before tourniquet deflation T4 = 5 minutes after deflation T5 = 15 minutes after deflation, T6 = 30 minutes after deflation.

EtCO_2_ = end-tidal carbon dioxide, HR = heart rate, MAP = mean arterial pressure, NIRS = near-infrared spectroscopy, ONSD = optic nerve sheath diameter.

* Statistically significantly lower compared to T0.

† Statistically significantly higher compared to T0.

‡ Statistically significantly lower compared to T1.

§ Statistically significantly higher compared to T1.

∥ Statistically significantly lower compared to T3.

¶ Statistically significantly higher compared to T3.

In the perioperative stage, the rSO_2_ values did not decrease below 50% in any of the patients. The right rSO_2_ values of the patients after intubation and before tourniquet inflation were higher than those measured before induction (*P* < .05). The right rSO_2_ values obtained after tourniquet deflation were higher than those measured before induction (*P* < .001). The right rSO_2_ values measured after tourniquet deflation were higher than those noted before tourniquet inflation and before tourniquet deflation (*P* < .05). The left rSO_2_ values of the patients measured after intubation were greater than their rSO_2_ values measured before induction (*P* < .001). The rSO_2_ values observed after tourniquet deflation were significantly higher than those measured before induction, before tourniquet inflation, and before tourniquet deflation (*P* < .05).

The ONSD values of the patients exceeded the 5 mm cutoff value only for the measurements taken at 5 minutes after tourniquet deflation (T4). The patients were therefore divided into 2 groups, that is, those with an ONSD <5 mm (n = 20) and those with an ONSD ≥5 mm (n = 21), and examined at T4. No significant differences were noted between these groups in terms of patient age, sex, BMI, ASA risk classes, smoking status, MAP, or rSO_2_ values (*P* > .05). The ONSD ≥5 mm group had higher EtCO_2_ values and tourniquet times (*P* < .05) (Table 3).

**Table 3:** Comparison of certain parameters measured at the T4 time point between the ONSD <5 mm and ONSD ≥5 mm groups.

	ONSD < 5 mm (n = 21)	ONSD ≥ 5 mm (n = 20)	*P*
Age (yr)	42.71 ± 13.56	52.95 ± 19.23	.055
Gender (female/male)	10/11	9/11	.884
BMI (kg/m^2^)	27.37 ± 3.55	28.55 ± 3.50	.289
ASA (I/II)	5/16	5/15	.783
Current smoker (yes/no)	11/10	5/15	.140
Tourniquet time (min)	110.42 ± 6.69	115.30 ± 5.97	.019
MAP (mm Hg)	72.14 ± 6.55	72.05 ± 7.93	.968
EtCO_2_ (mm Hg)	40.23 ± 2.16	41.85 ± 2.83	.047
Right rSO_2_ (%)	70.04 ± 6.40	68.75 ± 8.60	.586
Left rSO_2_ (%)	67.95 ± 7.29	68.45 ± 8.73	.844

ASA = American Society of Anesthesiologists, BMI = body mass index, EtCO_2_ = end-tidal carbon dioxide, MAP = mean arterial pressure, ONSD = optic nerve sheath diameter, rSO_2_ = cerebral oxygen saturation.

**Figure 2.**
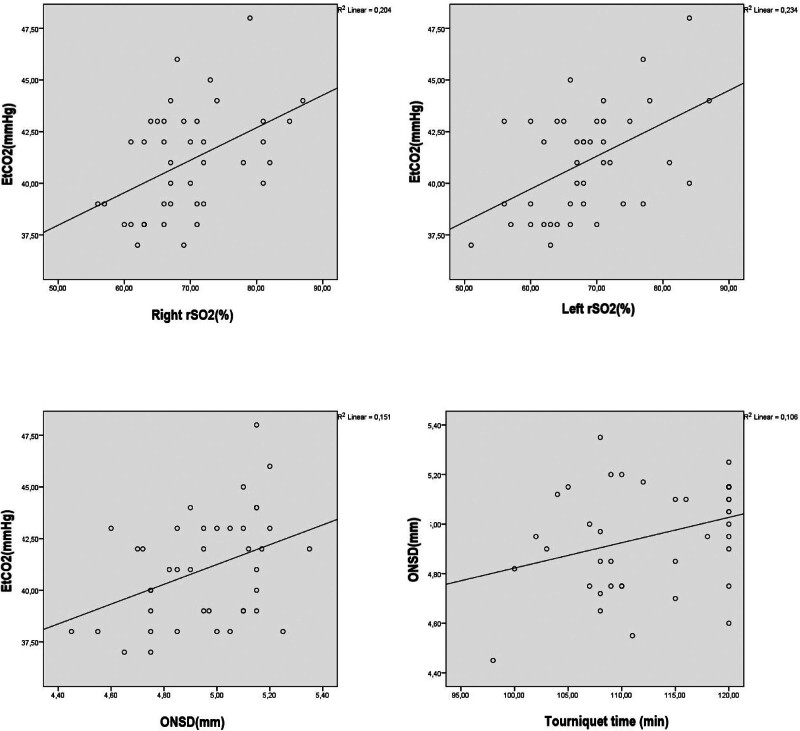
Correlations between the EtCO_2_ and ONSD values, between the ONSD values and tourniquet time, and between the EtCO_2_ and right/left rSO_2_ values. EtCO_2_ = end-tidal carbon dioxide, ONSD = optic nerve sheath diameter, rSO_2_ = cerebral oxygen saturation.

At T4, positive, moderate, and significant relationships were evident between the EtCO_2_ and ONSD values, between the ONSD values and tourniquet time, and between the EtCO_2_ and right/left rSO_2_ values (Fig. 2).

## 4. Discussion

In this study, which was performed to determine the effects of tourniquet application on ONSD and cerebral oxygenation in lower extremity surgeries, we observed that deflating the tourniquet resulted in significant increases in the ONSD and rSO_2_ values, and these increases were associated with the EtCO_2_ values and the duration of tourniquet application.

Lower extremity surgery constitutes an important part of orthopedic surgery and is usually performed using tourniquets.^[2]^ In addition to its advantages, such as the provision of a blood-free surgical site and limited transfusion requirements due to reduced blood loss, tourniquet application has disadvantages, such as pain, embolism, and hemodynamic and metabolic complications.^[1,2]^ Efforts to minimize these complications in surgeries where tourniquets are used are critically important for anesthesiologists. Research has been and continues to be conducted regarding the changes that occur during tourniquet application and following tourniquet deflation.

PaCO_2_ elevation is a complication that is seen following tourniquet deflation. With an increase in PaCO_2_, which is a strong vasoactive factor, cerebral vasodilation occurs, and cerebral blood volume increases. These increases result in an increase in ICP.^[3]^ It is known that PaCO_2_ values are correlated with EtCO_2_ values.^[11]^ Some published articles have shown that ICP increases correlate with EtCO_2_ increases after tourniquet deflation in lower extremity surgeries involving multitrauma patients, especially those with intracranial trauma.^[12,13]^ However, the methods described in these articles are invasive and difficult to practice and add costs. ICP measurement based on ONSD values has been used more frequently in recent years as a noninvasive method. Several clinical studies have shown a correlation between ONSD and ICP in both children and adults.^[14–16]^ In their systematic review and meta-analysis in which ONSD ultrasonography and computed tomography findings were compared, Ohle et al^[17]^ found that ONSD demonstrated excellent performance in detecting increased ICP, with a sensitivity of 95.6% and a specificity of 92.3%. Maissan et al^[18]^ compared invasive ICP measurements and ultrasonographic ONSD measurements taken before, during, and after tracheal aspiration, which is known to increase ICP, in patients with traumatic brain injury who were being monitored in the intensive care unit. The authors reported a correlation between the ONSD and intracranial measurements.

Tayal et al^[19]^ argued that to evaluate the increased ICP seen in the cranial computed tomography images of patients with acute head trauma accurately using ONSD measurements, it is necessary for physicians with bedside ultrasound scan experience to perform about 10 ultrasound scans with 3 abnormal results, while those with no such experience would need to perform 25 scans. Ballantyne et al^[20]^ stated that the mean interobserver variation in the ultrasonographic measurements of ONSD is ±0.2 mm, this measurement is an easy-to-learn and repeatable technique, and it is important to standardize these examination techniques. In our study, to achieve standardization, all the measurements were performed by the same physician, who had obtained experience with >25 ONSD measurements.

Kimberly et al,^[9]^ who examined the relationship between ONSD and ICP, reported that a cutoff value of ONSD > 5 mm was used successfully with 88% sensitivity and 93% specificity to detect ICP values higher than 20 cm H_2_O. To evaluate increased ICP, we also used an ONSD value of 5 mm as the cutoff value in the ultrasonographic ONSD measurements.

In a study in which they investigated the effects of tourniquet usage on ONSD, Beşir and Tuğcugil^[10]^ reported that the MAP values decreased below the baseline values after tourniquet deflation but returned to the values before tourniquet deflation after 10 minutes. They further found that the EtCO_2_ and ONSD values increased noticeably following tourniquet deflation and exceeded the 5 mm cutoff value, whereas they were higher than only the baseline values after 15 min. The authors concluded that the ONSD values correlated with the EtCO_2_ values after tourniquet deflation, and the increase in the ONSD values was dependent on the increase in the EtCO_2_ values in their study.

Kim et al^[21]^ investigated changes occurring in ICP as a result of tourniquet deflation using ONSD measurements in patients undergoing lower extremity surgeries and found that the change in the EtCO_2_ was similar to the change in the ONSD right before tourniquet deflation and up to 5 min after tourniquet deflation.

Venkateswarlu et al^[22]^ used ONSD to assess the ICP changes that occurred following tourniquet release in patients undergoing orthopedic surgery under spinal anesthesia. Among the measurements taken at 5, 10, and 15 minutes after tourniquet deflation, the authors detected the greatest degrees of change in the EtCO_2_ and ONSD values at 10 minutes.

Similar to the results of Beşir and Tuğcugil^[10]^ and Kim et al,^[21]^ we identified a significant relationship between the EtCO_2_ and ONSD values 5 minutes after tourniquet deflation. In contrast to this and the aforementioned studies, Venkateswarlu et al^[22]^ detected the greatest degrees of change in EtCO_2_ and ONSD at 10 minutes. We believe that this difference could be explained by their methodology, which involved the use of spinal anesthesia as the anesthesia method.

In this study, we observed that the EtCO_2_ values started to increase 60 minutes after tourniquet inflation, reached their maximum levels 5 minutes after tourniquet deflation, and returned to the values before deflation at 30 minutes. The ONSD values were higher than the baseline throughout the surgery, increased even further at 30 minutes after tourniquet inflation, and reached their maximum levels at 5 minutes following tourniquet deflation. At 5 minutes after tourniquet deflation, the ONSD values exceeded the 5 mm cutoff point in 51.2% of the patients. At 30 minutes after tourniquet deflation, these values returned to the levels measured immediately before deflation. In our study, the EtCO_2_ and ONSD values correlated, and the ONSD values increased depending on the increases in the EtCO_2_ values. For the measurements made 5 minutes after tourniquet deflation in the patients whose ONSD values were higher than the 5 mm cutoff value, we found that the patients had higher EtCO_2_ values and longer tourniquet application times.

Depending on the length of the tourniquet application time, various complications may develop. It has been shown that time-dependent progressive acidosis could develop in the venous blood at the distal end of the tourniquet. It is therefore recommended that the tourniquet time be kept to a minimum, with an upper limit of 2 hours in healthy patients.^[1]^

In a study in which they investigated the effects of tourniquet time and pressure on changes in ICP following tourniquet deflation, Beşir and Tuğcugil^[23]^ showed a positive significant correlation between tourniquet time and ONSD. When they used an ONSD ≥5 mm as the standard criterion, they found that the safe cutoff value for the optimal tourniquet time was <67.5 min (sensitivity: 87%, specificity: 59.5%). In our study, the mean tourniquet time in the group with an ONSD ≥5 mm was 115.30 ± 5.97 minutes, which was longer than the mean time in the group with an ONSD <5 mm. The tourniquet times in our study were thus longer than the cutoff value reported by Beşir and Tuğcugil. The increase in ONSD values in our study may have occurred as a result of acidosis depending on the increased PaCO_2_ and lactate levels that occurred in relation to the longer tourniquet times.

NIRS is a monitoring method designed to noninvasively and continuously estimate rSO2. rSO2 monitoring accurately reflects cerebral oxygenation during hypoxemia, hypocapnia, hypercapnia, and hypotension. Given this feature, it plays an important role in central nervous system monitoring, especially during surgery and anesthesia involving hypotensive patients.^[24]^ In particular, hypotension that develops after tourniquet deflation, along with surgical bleeding, can lead to cerebral hypoperfusion.^[25]^ It has been reported that NIRS could be effective in detecting cerebral hypoperfusion by offering an effective monitoring method for brain ischemia.^[6]^

Kim et al^[26]^ studied the effects of EtCO_2_ values on cerebral oxygen saturation in patients in the deckchair position under general anesthesia and reported a linear relationship between the EtCO_2_ and rSO_2_ values. Ay et al^[27]^ retrospectively examined the cerebral oxygenation monitoring processes of patients undergoing gynecologic laparoscopy procedures. The authors noted that the rSO_2_ values increased during the intraoperative stage, and this increase could have stemmed from an increase in the EtCO_2_ values that developed as a result of CO_2_ insufflation. Noticeable elevations were seen in the right and left rSO_2_ values in our study following tourniquet deflation. In the same measurement period, the MAP values dropped substantially while the EtCO_2_ values increased considerably. We opine that the increase in rSO_2_ values was caused by the increase in EtCO_2_ values.

The most important limitation of our study is that we did not measure PaCO_2_, which is an important indicator of cerebral blood flow. Because arterial cannulation is not a routine practice used in our patients, we used EtCO_2_ values, which are correlated with PaCO_2_. The lack of a global cerebral blood flow measurement to determine cerebral oxygenation may be considered another limitation of this study.

## 5. Conclusion

This prospective observational study demonstrated that tourniquet deflation during lower extremity surgery is associated with significant increases in ONSD and rSO_2_. These changes were significantly associated with elevations in EtCO_2_ levels and with tourniquet duration. While the findings highlight important physiological relationships between tourniquet deflation, P_a_CO_2_ levels, and surrogate markers of intracranial pressure and cerebral oxygenation, the clinical implications of these changes remain uncertain. Further studies incorporating direct intracranial pressure measurements and clinical neurological outcome parameters are required to clarify their clinical significance.

## Acknowledgments

This study was supported by the Scientific Research Projects Unit of Zonguldak Bülent Ecevit University (project no. 2019-52053101-03).

## Author contributions

**Funding acquisition:** Bahar Aktaş, Rahşan Dilek Okyay.

**Investigation:** Bahar Aktaş.

**Methodology:** Bahar Aktaş, Rahşan Dilek Okyay, Özcan Pişkin, Bengü Gülhan Köksal İncegül, Gamze Küçükosman, Eren Açikgöz, Çağdaş Baytar, Keziban Bollucuoğlu, Hilal Ayoğlu.

**Project administration:** Bahar Aktaş, Rahşan Dilek Okyay.

**Resources:** Bahar Aktaş, Rahşan Dilek Okyay, Bengü Gülhan Köksal İncegül, Gamze Küçükosman, Çağdaş Baytar, Hilal Ayoğlu.

**Software:** Bahar Aktaş, Rahşan Dilek Okyay, Bengü Gülhan Köksal İncegül, Çağdaş Baytar, Hilal Ayoğlu.

**Supervision:** Bahar Aktaş, Rahşan Dilek Okyay.

**Validation:** Bahar Aktaş, Rahşan Dilek Okyay, Bengü Gülhan Köksal İncegül, Çağdaş Baytar, Hilal Ayoğlu.

**Visualization:** Bahar Aktaş, Rahşan Dilek Okyay, Hilal Ayoğlu.

**Writing – original draft:** Bahar Aktaş, Rahşan Dilek Okyay.

**Writing – review & editing:** Bahar Aktaş, Rahşan Dilek Okyay, Özcan Pişkin, Bengü Gülhan Köksal İncegül, Gamze Küçükosman, Çağdaş Baytar, Keziban Bollucuoğlu, Hilal Ayoğlu.
